# The SMILES trial: do undisclosed recruitment practices explain the remarkably large effect?

**DOI:** 10.1186/s12916-018-1221-5

**Published:** 2018-12-28

**Authors:** Marc L. Molendijk, Eiko I. Fried, Willem Van der Does

**Affiliations:** 1 Institute of Psychology, Clinical Psychology Unit, Leiden University, Leiden, The Netherlands; 2 Leiden Institute of Brain and Cognition, Leiden University Medical Center, Leiden, The Netherlands; 3 Department of Psychology, University of Amsterdam, Amsterdam, The Netherlands; 4 Department of Psychiatry, Leiden University Medical Center, Leiden, The Netherlands

## Abstract

The SMILES trial showed substantial improvement of depressive symptoms following seven consultations on healthy dieting. The very large effect size on depression reduction seems remarkable and we suggest that selectively induced expectancy and a loss of blinding have contributed to the observed effect.

The recently published SMILES study [1] is the first randomized controlled trial (RCT) to assess the efficacy of a manipulated diet on depression, extending previous studies on single nutrients. The authors conclude that “*… dietary improvement guided by a clinical dietician may provide an efficacious treatment strategy for the management of this highly prevalent mental disorder*” ([1], p. 1), and that “*clinicians should* […] *consider promoting the benefits of dietary improvement and facilitating access to dietetics support for their patients with depression*” ([1], p. 11). At the same time, the authors acknowledge the need for replication while referring to the small sample size of their study (*N* = 56; about one-third of the sample size pre-specified as required for adequate power).

Studying the effects of diet on depression is of considerable public interest [1–4], and we agree that the topic warrants attention. However, we argue below that the results of the SMILES study should not be taken as evidence for diet efficacy given various issues regarding study recruitment and blinding. In the SMILES study, participants were randomized to seven sessions of either dietary support or social support. The dietary condition outperformed the control condition (social support) with an effect size (Cohen’s *d*) of 1.2, which is four to five times larger than established depression treatments (e.g., cognitive behavioral therapy, *d* = 0.2–0.3 [5]; antidepressants, *d* = 0.3 [6, 7]). The pre–post effect size of the diet condition was very large (*d* = 1.98), whereas the social support condition had an effect size of *d* = 0.63.

Since its publication, the SMILES trial has received considerable attention [8, 9], likely due to the very large effect and the conclusion from SMILES’ senior author in a follow-up article that the trial provides “*… intervention evidence supporting causality* [for dietary improvement as a treatment strategy for major depressive episodes]” ([4], p. 26). This aligns with other work by some of the SMILES trial team members in which diet and nutrition are conceptualized as a “*… central determinant of mental health*” ([2], p. 271).

The primary outcome in the SMILES trial was change in the severity of depression symptoms, in this case assessed via the interviewer-rated Montgomery–Åsberg Depression Rating Scale (MADRS) [10]. Such outcomes are subjective in nature (the participant is queried about symptoms), and thus susceptible to expectancy effects (defined as results biased by the communication of the expected results [11–14]). When the intervention is non-pharmacological – herein the advice of following a healthy diet – the blinding of conditions in an RCT is hardly possible. In order to minimize expectancy bias, the authors describe that they masked the hypothesis and used a neutral recruitment strategy. Participants were approached (in social media, local newspapers, and radio interviews) with the following message: “*We are trialing the effect of an educational and counseling program focusing on diet that may help improve the symptoms of depression*” ([1], p. 2). The paper further states that “*several strategies were employed to reduce the risk of bias*” ([1], p. 5) and that “*significant effort was made to mask our hypothesis*” ([1], p. 10).

In this letter, we argue that there is a mismatch between the reported and the actual recruitment strategy, which becomes clear when comparing the reported and actual recruitment strategies. Through a hyperlink to an external website on the study’s recruitment website [15] (note that there are some differences with current and previous versions), potential participants are presented to a ‘fruit smiley’, composed out of two green apples as eyes, a mandarin as nose, and a banana with the edges pointing up as a smiling mouth [16]. Messages such as: “*Bananas look like a smile but can also help you smile because they contain tryptophan which is a mood stabilizer*” and “*Banana, brazil nuts, broccoli, they all have something in common apart from starting with the letter B. They all contain nutrients which can stabilize mood*” are presented alongside. Since no benefits of the control conditioning (befriending) were described, we think that this is anything but a neutral description, which has likely affected expectancy in participants. The study hypothesis was provided at recruitment to potential participants ‘on a platter’, so to speak. Website visitors also learned that “*the fear that we are eating our way to depression is prompting governments to take action*” [15]. This was accompanied by testimonials, such as “*The solution to my depression is good quality food*”. In a local newspaper article entitled ‘Diet, Depression, Hope’ [17], published in the recruitment catchment area at time of enrolment, the senior author wrote “*if you eat a healthier diet then it reduces your risk on depression*”. This article was accompanied by an invitation to participate in the trial and contact details. In summary, the report of the SMILES trial does not seem to adequately describe how study recruitment took place in practice.

The befriending condition had a four-time higher drop-out rate than the dietary condition and its pre–post effect size (*d* = 0.6) is lower than the effect size usually reported for pill–placebo (*d* = 0.9) [6, 7], likely due to the selective induction of expectancy. This implies that the control condition was not perceived as an equally attractive condition by many participants, indicating selection bias. Given that a purpose of randomization is to prevent this bias from happening [18], we argue that the SMILES trial has essentially lost some of the benefits of randomization and, further, that conclusions that can be drawn from RCTs do not directly follow from the SMILES trial.

The SMILES trial investigators discuss that, despite the use of blinded raters, blinding may not have been sufficient. Additionally, the data contains evidence that blinding was imperfect, yet this is not discussed in the article. The pre–post effect sizes of the self-report Hospital Anxiety and Depression Scale and the interviewer-based MADRS are equal in the control condition. However, in the dietary condition, interviewer-rated improvement is much higher than self-reported improvement (*d* = 0.8 vs. *d* = 0 in the control condition), suggesting a loss of blinding.

In sum, the limitations outlined above, along with what we consider a serious mismatch between the actual and published recruitment strategies [1], means that the SMILES trial does not meet all criteria for an RCT. It must be noted, however, that the notion that a healthy diet may benefit depression has been discussed in media prior to the SMILES trial, and not always with the most critical scientific eye (e.g., [19]). Accordingly, Jacka et al. [1] mention the existence of expectancy effects as a potential limitation in the interpretation of the trial results. Pre-existing expectancies make any trial more difficult and this is certainly not the responsibility of the SMILES investigators, yet what we do consider their responsibility is a correct description of the study procedures. We believe that the SMILES trial falls short in this regard.
